# Deformation, fracture characteristics and damage constitutive model of soft coal under true triaxial complex stress paths

**DOI:** 10.1371/journal.pone.0319706

**Published:** 2025-02-27

**Authors:** Chongyang Jiang, Lianguo Wang, Jiaxing Guo, Shuai Wang

**Affiliations:** State Key Laboratory of Intelligent Construction and Healthy Operation and Maintenance of Deep Underground Engineering, China University of Mining and Technology, Xuzhou, Jiangsu, China; University of Science and Technology Beijing, CHINA

## Abstract

Understanding the mechanical properties and damage deterioration mechanisms of soft coal under true triaxial complex stress paths is crucial for predicting and evaluating the stability of the roof during roadway excavation in thick soft coal seams. This study examines the evolution of deformation strength, fracture characteristics, and acoustic emission patterns of soft coal under various initial stress levels and stress paths using true triaxial loading and unloading tests. The research reveals that soft coal undergoes rapid expansion deformation and ultimately fails along the unloading direction, which varies with different stress paths. The initial stress level and stress path significantly influence deformation and strength, conforming to the Mogi-Coulomb criterion. The fracture modes of the coal under different stress paths can be categorized into compressive-shear failure and compression-shear and tension composite failure. Furthermore, based on the experimental results, a damage constitutive model for soft coal is developed that integrates damage mechanics, Weibull statistical distribution theory, and the Mogi-Coulomb criterion to effectively measure microelement strength under true triaxial complex stress paths. Comparing the theoretical model with the experimental curves demonstrates that the proposed damage constitutive model can effectively reflect the deformation strength characteristics of soft coal under true triaxial complex stress paths. These findings offer a crucial theoretical foundation for enhancing methods to predict and evaluate the stability of roadway roofs in soft coal seams, potentially improving safety and efficiency in mining operations.

## 1. Introduction

As coal mining advances to deeper levels, the geological conditions, particularly in thick soft coal seams, become more complex. The low strength and high fragmentation tendency of soft coal increase the risk of roof collapse and cave-ins during roadway excavation. The mechanical properties and stability of soft coal are critical for the safe and efficient excavation of roadways. Soft coal experiences true triaxial stress conditions [1–3], undergoing complex loading and unloading processes during roadway excavation [4,5]. These processes vary at different locations [6]. The mechanical properties and fracture evolution of soft coal under complex stress paths determine the stability of the roadway roof. Therefore, understanding the mechanical properties and damage mechanisms of soft coal under true triaxial complex stress paths is crucial for predicting and evaluating roadway roof stability, thereby preventing major accidents.

True triaxial loading and unloading tests are valuable for simulating the complex stress changes experienced during roadway excavation. Various studies have systematically investigated the mechanical behavior of coal and rock under these conditions by controlling stress changes in different directions. Previous studies have explored various aspects of true triaxial testing. For instance, studies have addressed unloading failure mechanisms [7] and fracture mode evolution [8,9] in various rock types, as well as methods to quantify macroscopic cracks and dissipated energy during deformation [10,11]. Research has also focused on rockburst simulations in heterogeneous rocks [12], strength and deformation behaviors of hard rocks under diverse stress paths [13–15], and the analysis of deformation parameters, energy distribution, and fracture characteristics of coal under different stress paths [16]. However, most experimental studies on true triaxial loading and unloading focus on hard rocks such as marble, granite, sandstone, and basalt, with relatively simple stress paths. In contrast, there is limited research on the mechanical behavior of soft coal under true triaxial complex stress paths. Given the distinct characteristics of soft coal and its importance in mining operations, there remains a critical need to investigate its behavior under true triaxial complex stress conditions.

To accurately predict the deformation and failure behavior of coal and rock under complex conditions, it is crucial to establish reasonable constitutive models [17–19]. Significant progress has been made in studying damage constitutive models for coal and rock, primarily focusing on hard rocks or specific loading conditions. For instance, damage factors such as dissipated energy have been utilized to construct constitutive models that account for residual strength and progressive failure [20]. Statistical approaches have also been employed to capture anisotropic behavior under true triaxial stress conditions [21,22], while micro-mechanical theories have linked microcrack development with macroscopic deformation characteristics [23–25], enabling predictions for rock behavior under uniaxial or triaxial conditions. True triaxial tests have further provided critical data for refining these models, particularly in hard rocks. Key contributions include exploring the effects of loading rates [26], intermediate principal stress [27–29], and damage thresholds [30,31] which have been systematically incorporated into constitutive models for hard rock types. Despite these advancements, applying these insights to soft coal remains challenging due to its unique mechanical properties and complex stress responses. This study aims to fill this gap by establishing a damage constitutive model for soft coal under true triaxial complex stress paths.

Therefore, this paper investigates the deformation strength, fracture characteristics, and acoustic emission patterns of soft coal under various stress paths using true triaxial tests. A new damage constitutive model for soft coal under complex stress conditions is developed, shedding light on its damage and fracture mechanisms. These findings offer a crucial theoretical foundation for enhancing predictions and evaluations of roadway roof stability in soft coal seams.

## 2. Test methods

### 2.1 Sample preparation

**Fig 1:**
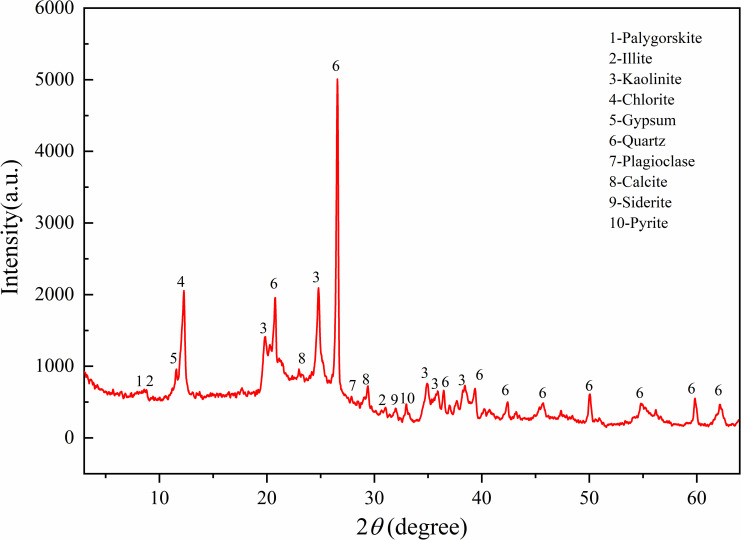
Raw coal XRD analysis spectrum.

**Fig 2:**
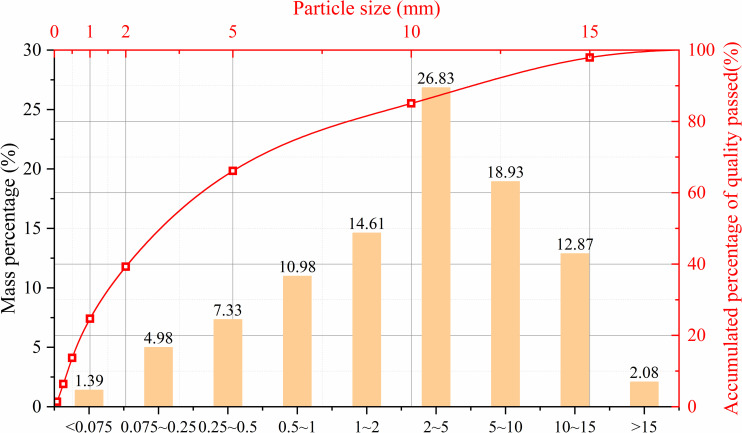
Raw coal particle size distribution.

The coal samples used in this experiment were taken from the No. 8 coal seam in the Huaibei mining area of China. This coal seam is extremely soft and easily fragmented, with very low strength, making sampling extremely difficult and unsuitable for direct processing into test samples. Therefore, equivalent soft coal samples were prepared from the loose raw coal collected on-site for the experiments. Coal is composed of various minerals, and the types and contents of these minerals determine whether the soft coal material can be formed into sample briquettes. First, an XRD phase diffraction analysis was conducted on the raw coal obtained on-site, and the relative contents of the whole rock minerals and clay minerals were determined through analysis. The results are shown in Fig 1 and Table 1, respectively. As seen from the figures, the main component of the raw coal is organic matter, with a relatively high content of clay minerals, primarily kaolinite. Kaolinite has plasticity and adhesion when wet, acting as a binder between coal particles, which is conducive to the formation of soft coal samples. Therefore, to replicate the actual bonding method of soft coal, no additional binder was added during sample preparation. The moisture content and particle size distribution of the raw coal were determined, with the average moisture content measured at 3.25%. The proportion of coal particles of different sizes and the particle size distribution curve of the raw coal are shown in Fig 2. It can be seen that the proportion of raw coal particles with a size of 0-15mm reached 97.92%, with blocks larger than 15mm accounting for only 2.08% of the total mass, primarily consisting of small gangue blocks, which are not representative. Therefore, raw coal particles sized 0-15mm were used for sample preparation in this experiment.

**Table 1:** Raw coal mineral content.

Whole Rock Minerals	Gypsum	Quartz	Plagioclase	Calcite	Siderite	Pyrite	Amorphous	Total Clay
Relative Content (%)	0.9	7.1	0.4	0.7	0.4	0.6	80.1	9.8
**Clay Minerals**	**Palygorskite**	**I/S Mixed Layer**	**Chlorite**	**Kaolinite**	**Illite**			
Relative Content (%)	16.5	2.4	10.1	64.1	6.9			

**Fig 3:**
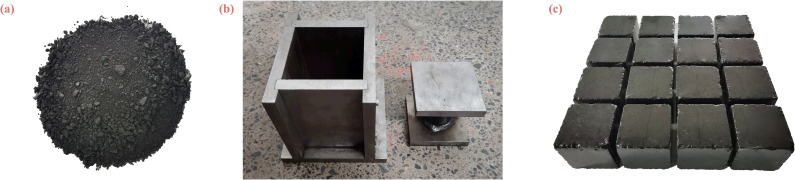
Coal briquette mold and samples. (a) Raw coal. (b) Sample mold. (c) Some coal briquettes.

The actual burial depth of the loose soft coal used in this experiment is about 850m, so the forming pressure of the coal briquettes was set to 22MPa. Based on multiple preliminary tests, it was determined that a total of 1480g of loose raw coal is required to prepare a coal sample. According to the particle size distribution of the raw coal, coal particles from each size range were weighed and then uniformly mixed (Fig 3a) before being added into the mold (Fig 3b). The testing machine was used to slowly apply pressure to the molding pressure of 22MPa, maintained for 20 minutes before the sample was removed, resulting in cubic samples with dimensions of 100mm × 100mm × 100mm, as shown in Fig 3c. The prepared samples were wrapped in plastic wrap for storage. To ensure sample homogeneity, ultrasonic velocity tests were conducted, and samples with high velocity variability were excluded. The selected samples had velocities ranging from 0.279km/s to 0.364km/s, with an average velocity of 0.331km/s.

### 2.2 Test equipment

**Fig 4:**
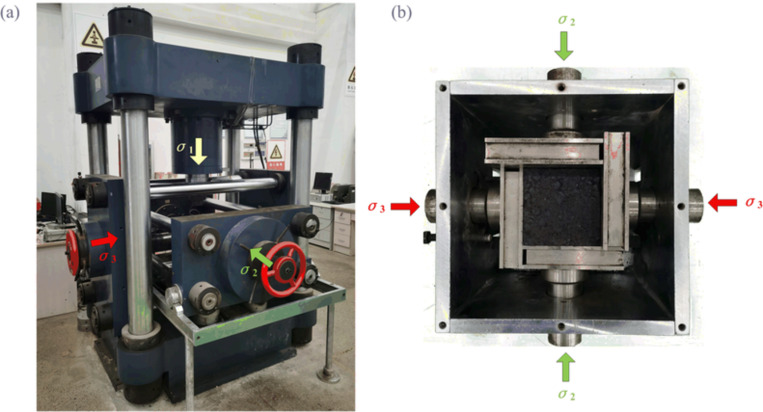
True triaxial electro-hydraulic servo loading test system. (a) True triaxial test machine. (b) True triaxial pressure chamber.

The test utilized the rock true triaxial electro-hydraulic servo loading test system of China University of Mining and Technology. The true triaxial test system mainly comprises a triaxial servo-controlled loading system, automatic acquisition system, true triaxial pressure chamber, and acoustic emission monitoring system [2]. As shown in Fig 4a, the triaxial servo-controlled loading system consists of three mutually perpendicular and independent loading subsystems ($\sigma_{1}$ , $\sigma_{2}$ and $\sigma_{3}$). Each servo control is independent, adopting a rigid loading method with maximum loading capacities of 1600 kN, 500 kN, and 300 kN, respectively. The force measurement accuracy is 0.01kN, and the deformation measurement accuracy is 0.002mm. As shown in Fig 4b, the true triaxial pressure chamber consists of a pressure box, base, loading plates, etc., located at the center of the triaxial servo-controlled loading system. The samples and loading plates adopt an “interlocking” arrangement to achieve loading in the three principal stress directions. During the test, the acoustic emission monitoring system was used to record the acoustic emission activity patterns of the soft coal during the true triaxial loading and unloading process.

### 2.3 Test plan

During the excavation of roadways, the stress state of the coal body ahead of the excavation face continuously changes, leading to stress concentration, stress unloading, and simultaneous stress concentration and unloading in different directions. To study the deformation strength evolution and fracture patterns of soft coal under different stress states and paths, this experiment designed true triaxial loading and unloading tests with 6 stress paths under 3 initial stress levels. The test plan is shown in Table 2.

**Table 2:** Loading and unloading test plan for soft coal under complex stress paths.

Test Type	Initial Stress Level/MPa			Stress Path	Test Number
	$\sigma_{1}$	$\sigma_{2}$	$\sigma_{3}$		
Constant axial compression and unloading confining pressure	20	15	10	Path 1: Keep $\sigma_{1}$ constant, unload $\sigma_{3}$	C1-U3-1
	25	20	10		C1-U3-2
	30	25	10		C1-U3-3
	20	15	10	Path 2: Keep $\sigma_{1}$ constant, unload $\sigma_{2}$	C1-U2-1
	25	20	10		C1-U2-2
	30	25	10		C1-U2-3
	20	15	10	Path 3: Keep $\sigma_{1}$ constant, unload $\sigma_{2}$, $\sigma_{3}$	C1-U2U3-1
	25	20	10		C1-U2U3-2
	30	25	10		C1-U2U3-3
Load axial compression and unload confining pressure	20	15	10	Path 4: Load $\sigma_{1}$, unload $\sigma_{3}$	L1-U3-1
	25	20	10		L1-U3-2
	30	25	10		L1-U3-3
	20	15	10	Path 5: Load $\sigma_{1}$, unload $\sigma_{2}$	L1-U2-1
	25	20	10		L1-U2-2
	30	25	10		L1-U2-3
	20	15	10	Path 6: Load $\sigma_{1}$, unload $\sigma_{2}$, $\sigma_{3}$	L1-U2U3-1
	25	20	10		L1-U2U3-2
	30	25	10		L1-U2U3-3

(1) Initial Stress Loading Phase

First, the coal sample is loaded to the preset initial true triaxial stress state. Force control is used to load $\sigma_{1}$, $\sigma_{2}$ and $\sigma_{3}$ simultaneously to the target hydrostatic pressure state (10MPa) at a loading rate of 0.2MPa/s. Then, keeping $\sigma_{3}$ constant, $\sigma_{2}$ is loaded to the test set value at a rate of 0.2MPa/s. Finally, keeping $\sigma_{2}$ and $\sigma_{3}$ constant, $\sigma_{1}$ is loaded to the test set value at a rate of 0.2MPa/s, and maintained to achieve the initial true triaxial stress state.

(2) Loading and Unloading Phase with Different Stress Paths

**Fig 5:**
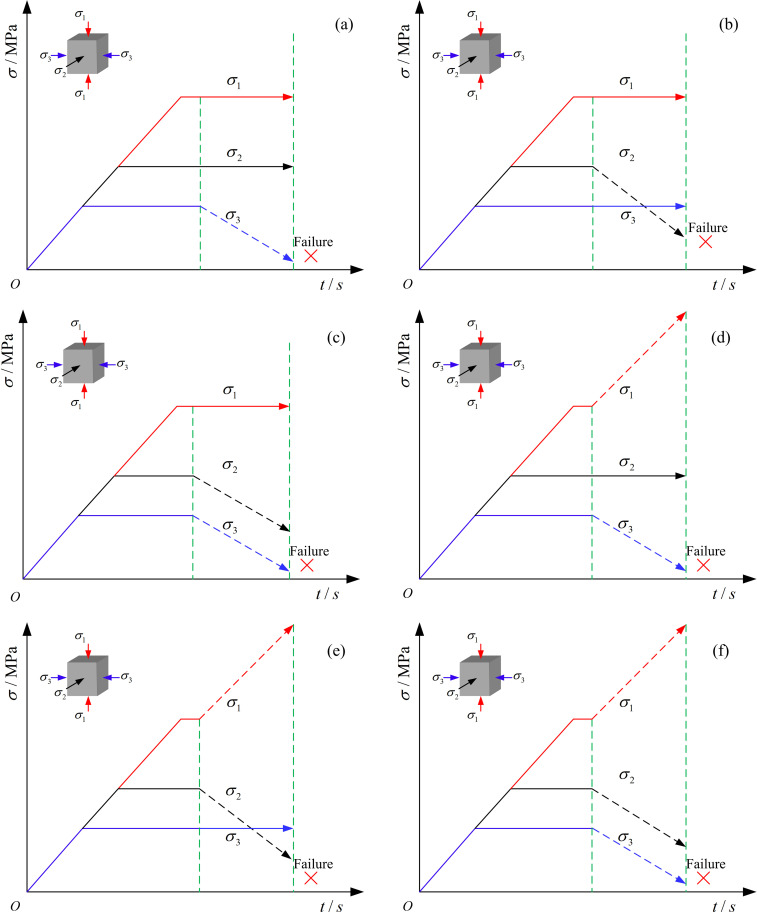
Schematic diagram of different loading and unloading stress paths. (a) Path 1. (b) Path 2. (c) Path 3. (d) Path 4. (e) Path 5. (f) Path 6.

After completing the initial stress loading, loading and unloading tests are conducted according to different stress paths. Fig 5 shows six stress loading and unloading paths. Paths 1 to 3 are constant axial compression and unloading confining pressure tests, while paths 4 to 6 are loading axial compression and unloading confining pressure tests.

Path 1: Keeping $\sigma_{1}$ and $\sigma_{2}$ constant, unload $\sigma_{3}$ at a rate of 0.1MPa/s until the coal sample fails.
Path 2: Keeping $\sigma_{1}$ and $\sigma_{3}$ constant, unload $\sigma_{3}$ at a rate of 0.1MPa/s until the coal sample fails.
Path 3: Keeping $\sigma_{1}$ constant, simultaneously unload $\sigma_{2}$ and $\sigma_{3}$ at a rate of 0.1MPa/s until the coal sample fails.
Path 4: Keeping $\sigma_{2}$ constant, load $\sigma_{1}$ at a rate of 0.1MPa/s while unloading $\sigma_{3}$ at a rate of 0.1MPa/s until the coal sample fails.
Path 5: Keeping $\sigma_{3}$ constant, load $\sigma_{1}$ at a rate of 0.1MPa/s while unloading $\sigma_{2}$ at a rate of 0.1MPa/s until the coal sample fails.
Path 6: Load $\sigma_{1}$ at a rate of 0.1MPa/s while simultaneously unloading $\sigma_{2}$ and $\sigma_{3}$ at a rate of 0.1MPa/s until the coal sample fails.

## 3. Analysis and discussion of test results

### 3.1 Deformation characteristics of soft coal under complex stress paths

Stress-strain curves are crucial for depicting the entire process of soft coal from crack initiation, crack propagation to failure and instability, and effectively reflecting the deformation and strength characteristics of soft coal. Therefore, obtaining stress-strain curves of soft coal under different stress path conditions is essential for studying the mechanical characteristics and failure properties of soft coal. For comparison and analysis, the deformation occurred when the initial stress loading in the three principal stress directions of the sample is completed is taken as the zero point, and only the data of the subsequent loading and unloading process are analyzed.

#### 3.1.1 Deformation characteristics of coal under constant axial compression and unloading confining pressure conditions.

**Fig 6:**
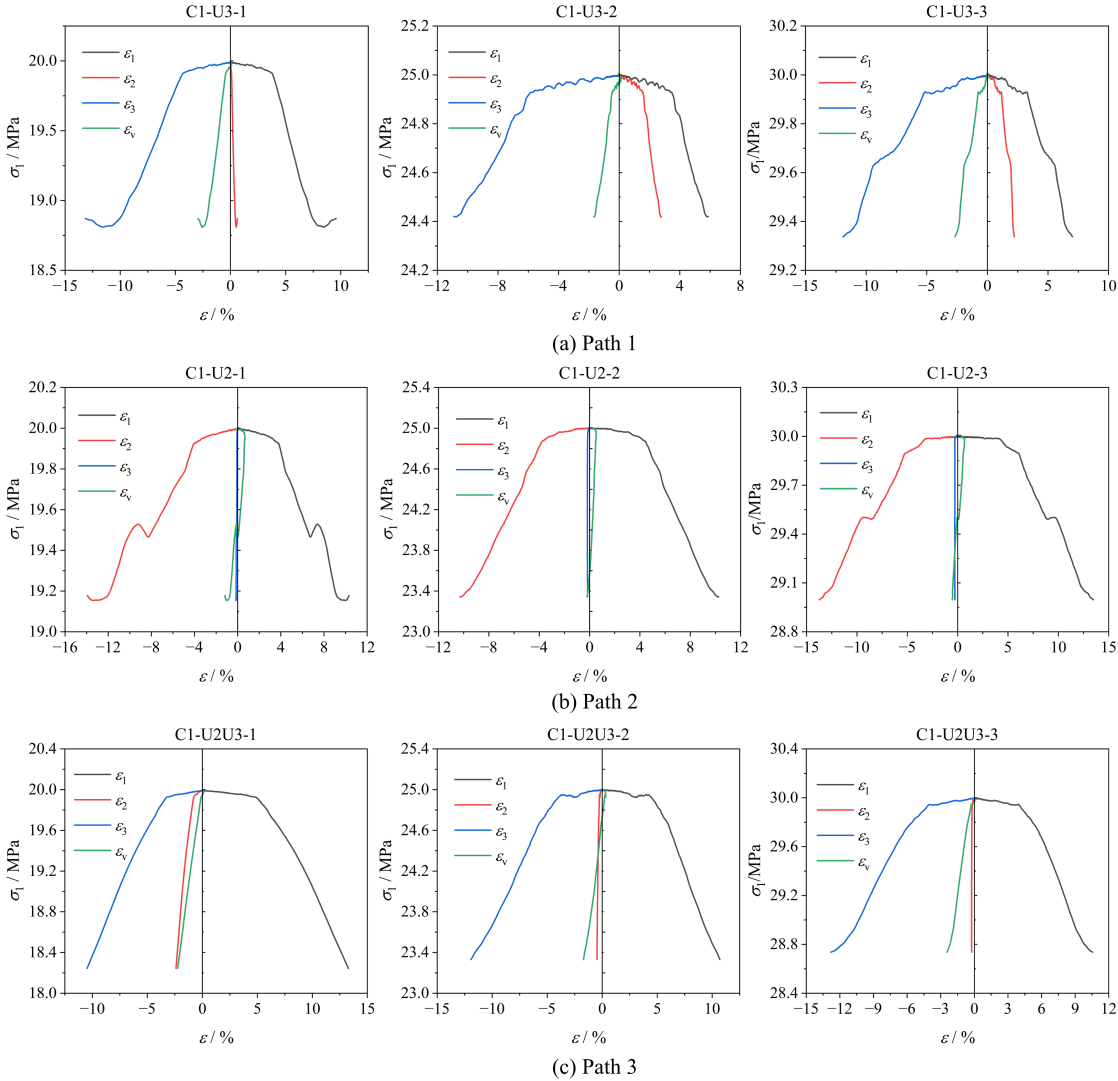
Stress-strain curve of soft coal under constant axial compression and unloading confining pressure conditions. (a) Path 1. (b) Path 2. (c) Path 3.

**Fig 7:**
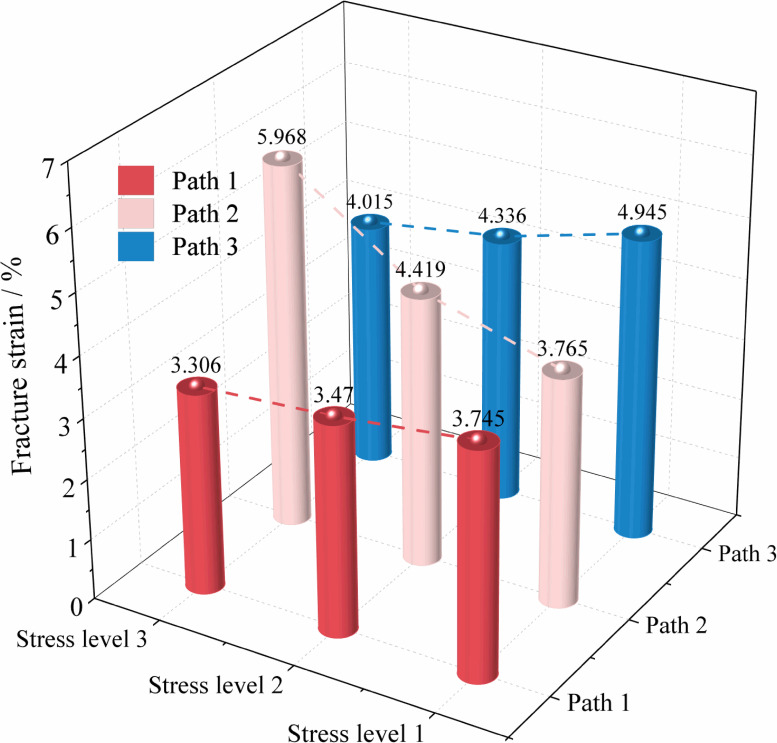
Strain statistics of soft coal at failure under constant axial compression and unloading confining pressure conditions.

Fig 6 shows the variation curves of axial stress ($\sigma_{1}$) with axial strain ($\varepsilon_{1}$), lateral strain ($\varepsilon_{2}$, $\varepsilon_{3}$), and volumetric strain ($\varepsilon_{\text{v}}$) of soft coal under constant axial compression and unloading confining pressure conditions. Fig 7 shows the strain variation patterns of soft coal under different stress paths during failure.

From Fig 6, it can be seen that under constant axial compression and unloading confining pressure conditions, the stress-strain curves of the three paths can be divided into two stages: the stable stage and the failure and instability stage. When the unloading begins, the soft coal sample first remains stable. As the lateral stress gradually decreases, and the coal body expands rapidly in the unloading direction, the volumetric strain $\varepsilon_{v}$ gradually decreases. The axial stress $\sigma_{1}$ gradually decreases until the coal fails and loses its load-bearing capacity. Under Path 1, as $\sigma_{3}$ unloads, $\varepsilon_{3}$ continuously decreases while $\varepsilon_{2}$ gradually increases, causing the coal body to compress in the $\sigma_{2}$ direction, promoting expansion deformation in the $\sigma_{3}$ direction and accelerating the coal failure, a phenomenon that becomes more evident as the initial stress level increases. Under Path 2, as $\sigma_{2}$ gradually decreases, $\varepsilon_{2}$ continuously decreases, causing the coal body to expand in the $\sigma_{2}$ direction until failure. Under Path 3, $\sigma_{2}$ and $\sigma_{3}$ decrease simultaneously, with $\varepsilon_{2}$ and $\varepsilon_{3}$ continuously decreasing, but the reduction rate of $\varepsilon_{3}$ is significantly higher than $\varepsilon_{2}$. This becomes more pronounced as the difference between initial $\sigma_{2}$ and $\sigma_{3}$ increases, causing the coal body to mainly undergo expansion failure in the $\sigma_{3}$ direction.

From Fig 7, it can be observed that under the same stress path conditions, the strain at failure of soft coal exhibits different trends as the initial stress level increases. Under Paths 1 and 3, the strain at failure gradually decreases with increasing initial stress levels. Specifically, the strain decreases by 7.34% and 11.72% under Path 1, and by 12.31% and 18.81% under Path 3. This indicates that higher initial stress levels in these paths promote failure instability in the coal. In contrast, under Path 2, the strain at failure increases with rising initial stress levels, showing increments of 17.37% and 58.51%. This suggests that in this case, higher initial stress levels enhance the load-bearing capacity of the coal. At the same stress level, the strain at failure under Path 1 is the smallest, primarily because in Path 1, as $\sigma_{3}$ unloads, $\sigma_{1}$ and $\sigma_{2}$ compress the sample together, accelerating the expansion failure of the coal in the $\sigma_{3}$ direction. Conversely, the strain at failure is largest under Path 5 due to the constraint imposed by the high $\sigma_{2}$ stress, which limits the expansion failure of the coal in that direction.

#### 3.1.2 Deformation characteristics of coal under loading axial compression and unloading confining pressure conditions.

**Fig 8:**
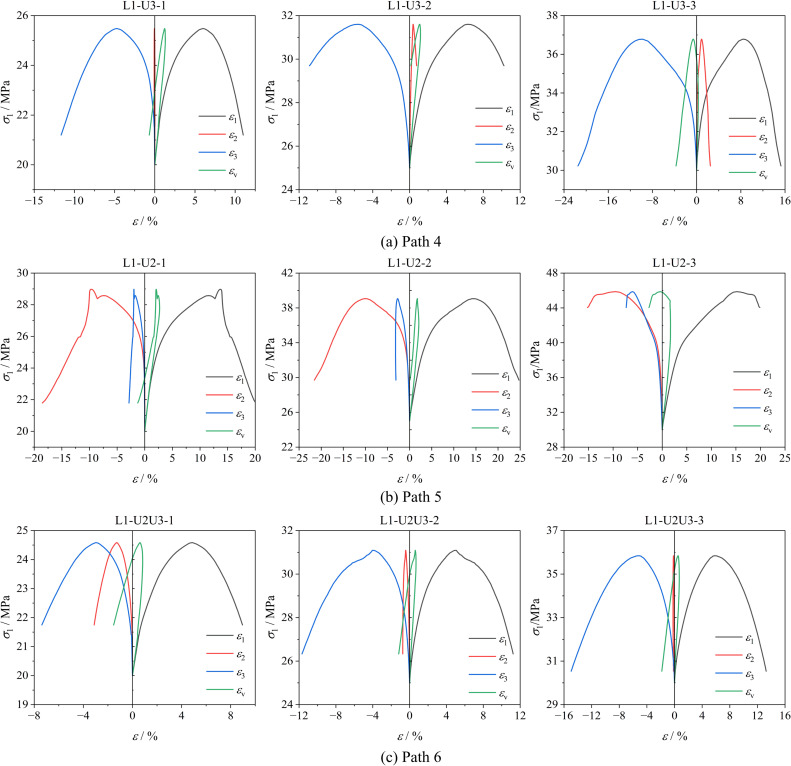
Stress-strain curve of soft coal under loading axial compression and unloading confining pressure conditions. (a) Path 4. (b) Path 5. (c) Path 6.

**Fig 9:**
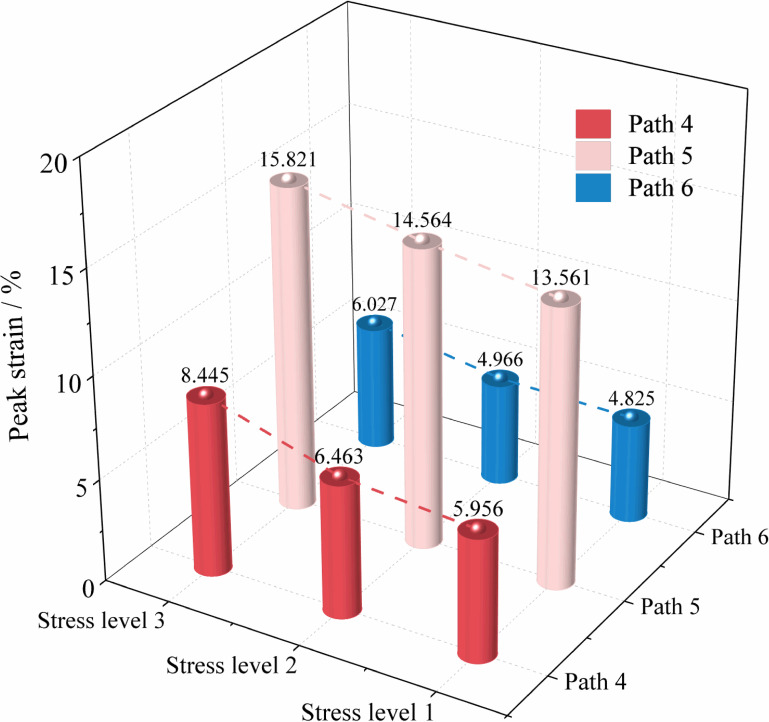
Statistics of peak strain of soft coal under loading axial compression and unloading confining pressure conditions.

Fig 8 shows the variation curves of axial stress ($\sigma_{1}$) with axial strain ($\varepsilon_{1}$), lateral strain ($\varepsilon_{2}$, $\varepsilon_{3}$), and volumetric strain ($\varepsilon_{\text{v}}$) of soft coal under loading axial compression and unloading confining pressure conditions. Fig 9 shows the variation patterns of peak strain of soft coal under different stress paths.

From Fig 8, it can be seen that under loading axial compression and unloading confining pressure conditions, the stress-strain curves of the three paths can also be divided into two stages: the pre-peak stage and the post-peak failure and instability stage. When loading axial compression and unloading confining pressure, the axial stress of the soft coal sample shows a nonlinear increase, with the rate of increase gradually slowing. After reaching the peak, the axial stress gradually decreases, while the strain in the unloading direction increases sharply, causing the coal body to expand rapidly in the unloading direction until failure. During this process, the volumetric strain first increases and then decreases, indicating that the volume change of the coal body is primarily axial compression in the initial loading and unloading stages, with lateral expansion gradually becoming dominant as the test proceeds. Under Path 4, the coal body shows a similar pattern to Path 1, with the coal body gradually compressing in the $\sigma_{2}$ direction, further promoting expansion deformation in the $\sigma_{3}$ direction and accelerating failure. Under Path 5, during the initial unloading phase, the coal body mainly undergoes expansion deformation in the $\sigma_{3}$ direction. As $\sigma_{2}$ gradually decreases to equal $\sigma_{3}$, the roles reverse, with the intermediate principal stress becoming the minimum principal stress. At this point, the reduction rate of $\sigma_{2}$ significantly exceeds that of $\varepsilon_{3}$, causing the coal body to begin expanding gradually in the $\sigma_{2}$ direction until failure. Under Path 6, as the confining pressure decreases, the reduction rate of $\varepsilon_{3}$ is significantly higher than that of $\varepsilon_{2}$, especially when the initial difference between $\sigma_{2}$ and $\sigma_{3}$ is large, indicating that the coal body mainly undergoes expansion failure in the $\sigma_{3}$ direction.

From Fig 9, it can be observed that under the same stress path, the peak strain of soft coal gradually increases with the rise in initial stress levels. Specifically, under Path 4, the peak strain increases by 8.51% and 41.79% as the stress level rises. Similarly, under Path 5, the peak strain increases by 7.39% and 16.67%, while under Path 6, the increases are 2.92% and 24.91%, respectively. At the same stress level, the peak strain of the coal body is the highest under Path 5, followed by Path 4, and the smallest under Path 6. This indicates that the coal body is least prone to failure and instability under Path 5. The reason is that, in the early stages of unloading $\sigma_{2}$, the coal expands along the $\sigma_{3}$ direction until $\sigma_{2}$ and $\sigma_{3}$ reverse roles, causing the coal to begin expanding along the $\sigma_{2}$ direction until failure occurs. During the early unloading, $\sigma_{2}$ provides a degree of protection to the coal body, enhancing its load-bearing capacity and thereby increasing the peak strain. In contrast, under Path 6, the coal sample is most susceptible to failure and instability, mainly because as $\sigma_{2}$ and $\sigma_{3}$ unload, the lateral constraint on the coal body gradually decreases, and the axial loading ($\sigma_{1}$) accelerates the lateral expansion deformation failure of the coal body, exacerbating crack propagation, reducing load-bearing capacity, and resulting in a lower peak strain.

### 3.2 Strength characteristics of soft coal under complex stress paths

#### 3.2.1 Applicability discussion of strength criteria.

From the commonly used strength criteria theories for coal and rock, the Mohr-Coulomb criterion, Drucker-Prager criterion, and Mogi-Coulomb criterion were selected to compare and analyze their applicability in the true triaxial complex stress path loading and unloading tests of soft coal.

(1) Mohr-Coulomb Criterion

The Mohr-Coulomb strength criterion is the most widely used strength theory in the geotechnical field [32]. Its expression is simple, and its physical meaning is clear. When expressed in terms of principal stress, it can be represented as:


$$\sigma_{1}=\frac{1+\sin\phi }{1-\sin\phi }\sigma_{3}+\frac{2c\cos\phi }{1-\sin\phi }$$ (1)

The yield function of the Mohr-Coulomb strength criterion can be expressed as:


$$f=(\sigma_{1}-\sigma_{3})-(\sigma_{1}+\sigma_{3})\sin\phi -2c\cos\phi$$ (2)

where *ϕ* is the internal friction angle of the coal and rock, *c* is the cohesion, and *f* is the yield function. *f* = 0 represents the yield surface in the principal stress space.

(2) Drucker-Prager Criterion

The Drucker-Prager criterion is a classical plastic theory model used to describe the plastic behavior of geomaterials such as rocks and soil under complex stress states. It is one of the important strength criteria in plastic mechanics [33]. The DP criterion considers the influence of the intermediate principal stress and the hydrostatic pressure, and can better predict the plastic yield and instability behavior of coal and rock under triaxial stress states. Its yield function can be expressed as:


$$f=\alpha I_{1}+\sqrt{J_{2}}-K$$ (3)

$$I_{1}=\sigma_{1}+\sigma_{2}+\sigma_{3}$$ (4)

$$J_{2}=\frac{1}{6}\left[{\left(\sigma_{1}-\sigma_{2}\right)}^{2}+{\left(\sigma_{1}-\sigma_{3}\right)}^{2}+{\left(\sigma_{2}-\sigma_{3}\right)}^{2}\right]$$ (5)

where $I_{1}$ is the first invariant of stress, $J_{2}$ is the second invariant of the deviatoric stress, and *α* and *K* are experimental constants related to the internal friction angle *ϕ* and cohesion *c* of the coal and rock, that is:


$$\{\begin{array}{l} \alpha =\frac{2\sin\phi }{\sqrt{3}(3-\sin\phi )}\\ K=\frac{6c\cos\phi }{\sqrt{3}(3-\sin\phi )} \end{array}$$ (6)

(3) Mogi-Coulomb Criterion

The Mogi-Coulomb criterion is an empirical criterion based on a large amount of true triaxial test data. This criterion assumes that the sample yields or fails when the octahedral shear stress $\tau_{\text{oct}}$ on any plane reaches a limit value [34,35]. It can be represented as:


$$\tau_{\text{oct}}=a+b\sigma_{m,2}$$ (7)

$$\tau_{\text{oct}}=\frac{1}{3}\sqrt{{\left(\sigma_{1}-\sigma_{2}\right)}^{2}+{\left(\sigma_{1}-\sigma_{3}\right)}^{2}+{\left(\sigma_{2}-\sigma_{3}\right)}^{2}}$$ (8)

$$\sigma_{m,2}=\frac{\sigma_{1}+\sigma_{3}}{2}$$ (9)

where $\tau_{\text{oct}}$ is the octahedral shear stress, $\sigma_{m,2}$ is the mean stress, and *a* and *b* are experimental constants related to the internal friction angle *ϕ* and cohesion *c* of the coal and rock, that is:


$$\{\begin{array}{l} a=\frac{2\sqrt{2}}{3}c\cos\phi \\ b=\frac{2\sqrt{2}}{3}\sin\phi \end{array}$$ (10)

**Fig 10:**
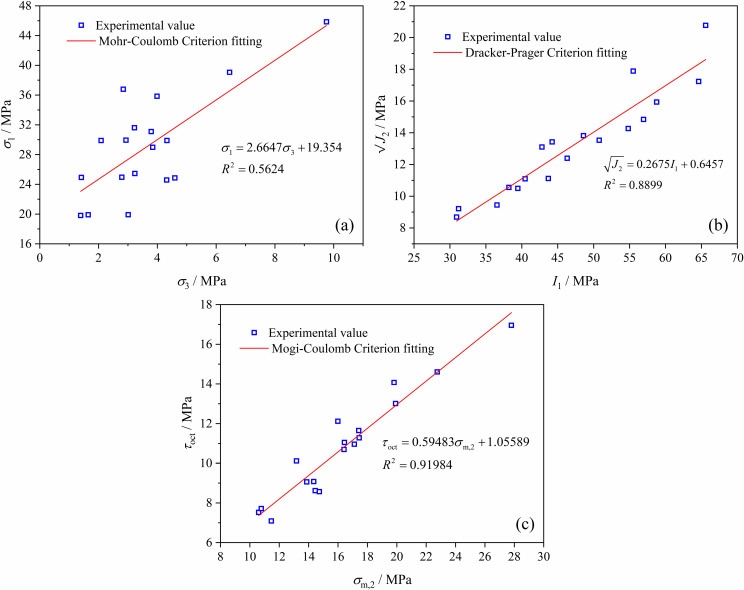
Fitting results of the three strength criteria. (a) Mohr-Coulomb. (b) Drucker-Prager. (c) Mogi-Coulomb.

**Fig 11:**
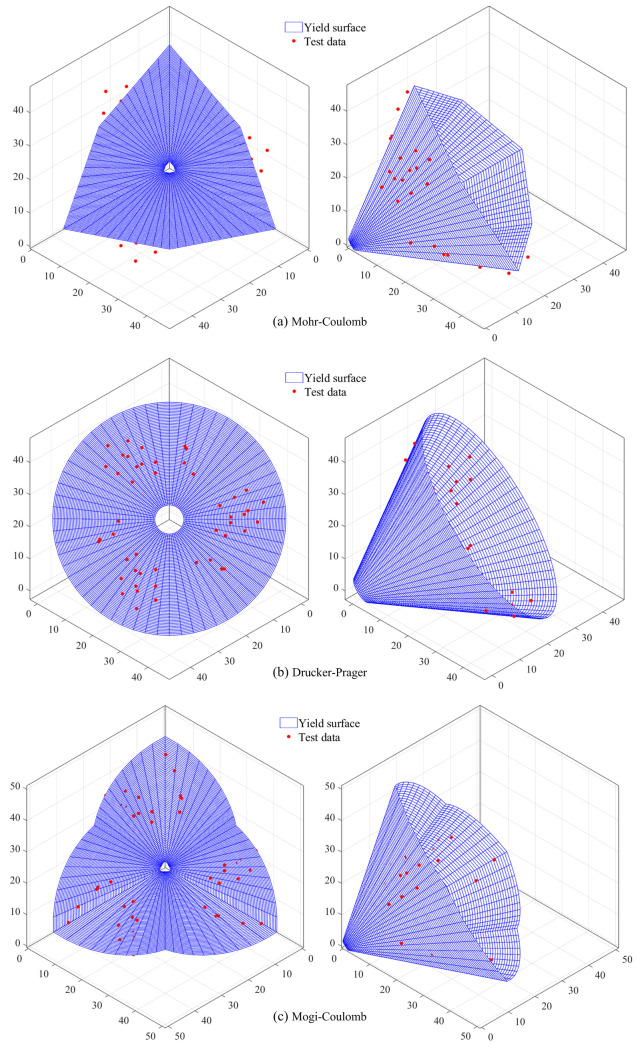
Yield surfaces of the three strength criteria. (a) Mohr-Coulomb. (b) Drucker-Prager. (c) Mogi-Coulomb.

Using the above three strength criteria, the strength of soft coal under different stress paths in the true triaxial tests was fitted, and the fitting results are shown in Fig 10. The yield surfaces of the three strength criteria and the position of each test data on the corresponding yield surface are shown in Fig 11.

According to the fitting results and yield surfaces shown in Figs 10 and 11, the fitting degree *R*^2^ using the Mohr-Coulomb strength criterion is only 0.56, with all test data located outside the yield surface. This indicates that the theoretical strength of the coal body is lower than the actual strength because the Mohr-Coulomb strength criterion does not consider the influence of the intermediate principal stress. Therefore, the Mohr-Coulomb strength criterion is not suitable for describing the strength characteristics of coal under complex true triaxial stress paths. When using the Drucker-Prager criterion to fit the test data, the fitting degree *R*^2^ reaches 0.89, with most test data located inside the yield surface, indicating that the theoretical strength of the coal body derived from the Drucker-Prager criterion is higher than the actual strength. When fitting with the Mogi-Coulomb criterion, the fitting degree *R*^2^ reaches 0.92, which is relatively high, and the test data are basically located on the theoretical yield surface. This indicates that the Mogi-Coulomb criterion is most suitable for describing the strength characteristics of soft coal under complex true triaxial stress paths.

#### 3.2.2 Strength evolution of coal under different stress paths.

**Fig 12:**
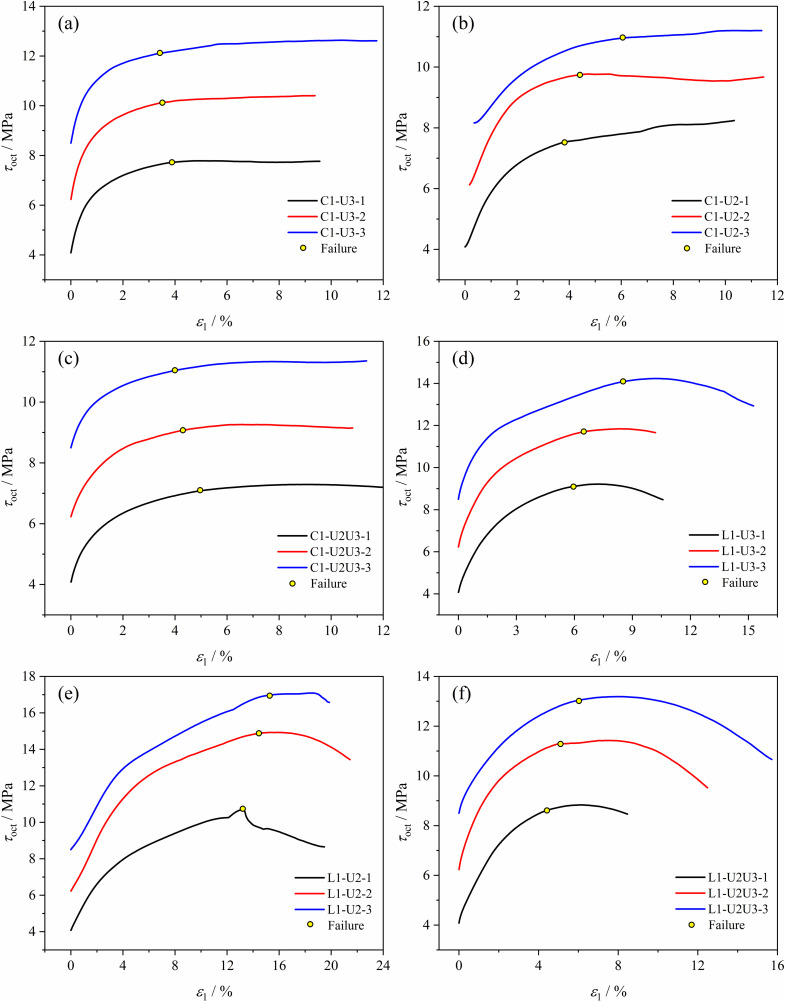
$\tau_{\text{oct}}-\varepsilon_{1}$ curves of soft coal under different stress paths. (a) Path 1. (b) Path 2. (c) Path 3. (d) Path 4. (e) Path 5. (f) Path 6.

**Fig 13:**
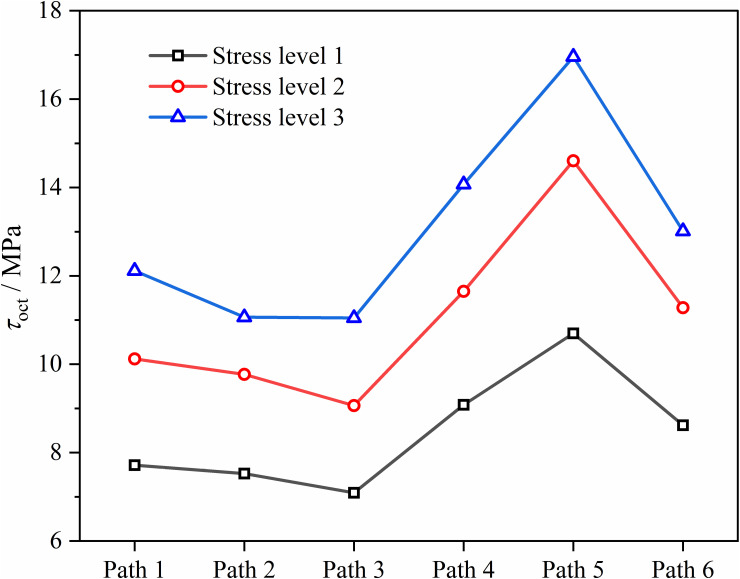
Variation of $\tau_{\text{oct}}$ at failure under different stress paths.

Due to the variation of the three principal stresses of soft coal during loading under different stress paths, to uniformly analyze the evolution of sample strength under different stress paths, the change curve of octahedral shear stress $\tau_{\text{oct}}$ with strain $\varepsilon_{1}$ was used, as shown in Fig 12. Fig 13 shows the variation of $\tau_{\text{oct}}$ at failure under different stress paths.

As shown in Fig 12, under the three paths of constant axial pressure and unloading confining pressure, $\tau_{\text{oct}}$ of the coal increases rapidly with $\varepsilon_{1}$ initially, and then $\tau_{\text{oct}}$ gradually stabilizes after the coal fails. Under the three paths of increasing axial pressure and unloading confining pressure, $\tau_{\text{oct}}$ first increases and then decreases, with the coal typically failing when $\tau_{\text{oct}}$ reaches its maximum value, further validating the applicability of the Mogi-Coulomb criterion. According to Fig 13, $\tau_{\text{oct}}$ at failure increases to varying degrees as the initial stress level rises under the same stress path. At the same initial stress level, $\tau_{\text{oct}}$ at failure is generally higher under conditions of increasing axial pressure and unloading confining pressure compared to constant axial pressure and unloading confining pressure, indicating that stress paths significantly affect the coal strength. The relationship is: Path 5>  Path 4>  Path 6>  Path 1>  Path 2>  Path 3. Under the three stress levels, Path 3 (constant $\sigma_{1}$, unloading $\sigma_{2}$ and $\sigma_{3}$) has the lowest $\tau_{\text{oct}}$ values at failure, being 7.09 MPa, 9.06 MPa, and 11.05 MPa, indicating that reducing lateral stress makes the sample more prone to failure. Path 5 (increasing $\sigma_{1}$, unloading $\sigma_{2}$) has the highest $\tau_{\text{oct}}$ values at failure, being 10.70 MPa, 14.61 MPa, and 16.96 MPa, because the difference between $\sigma_{2}$ and $\sigma_{3}$ gradually decreases and may even reverse, with $\sigma_{2}$ providing some protection to the coal.

### 3.3 Fracture characteristics of soft coal under complex stress paths

**Fig 14:**
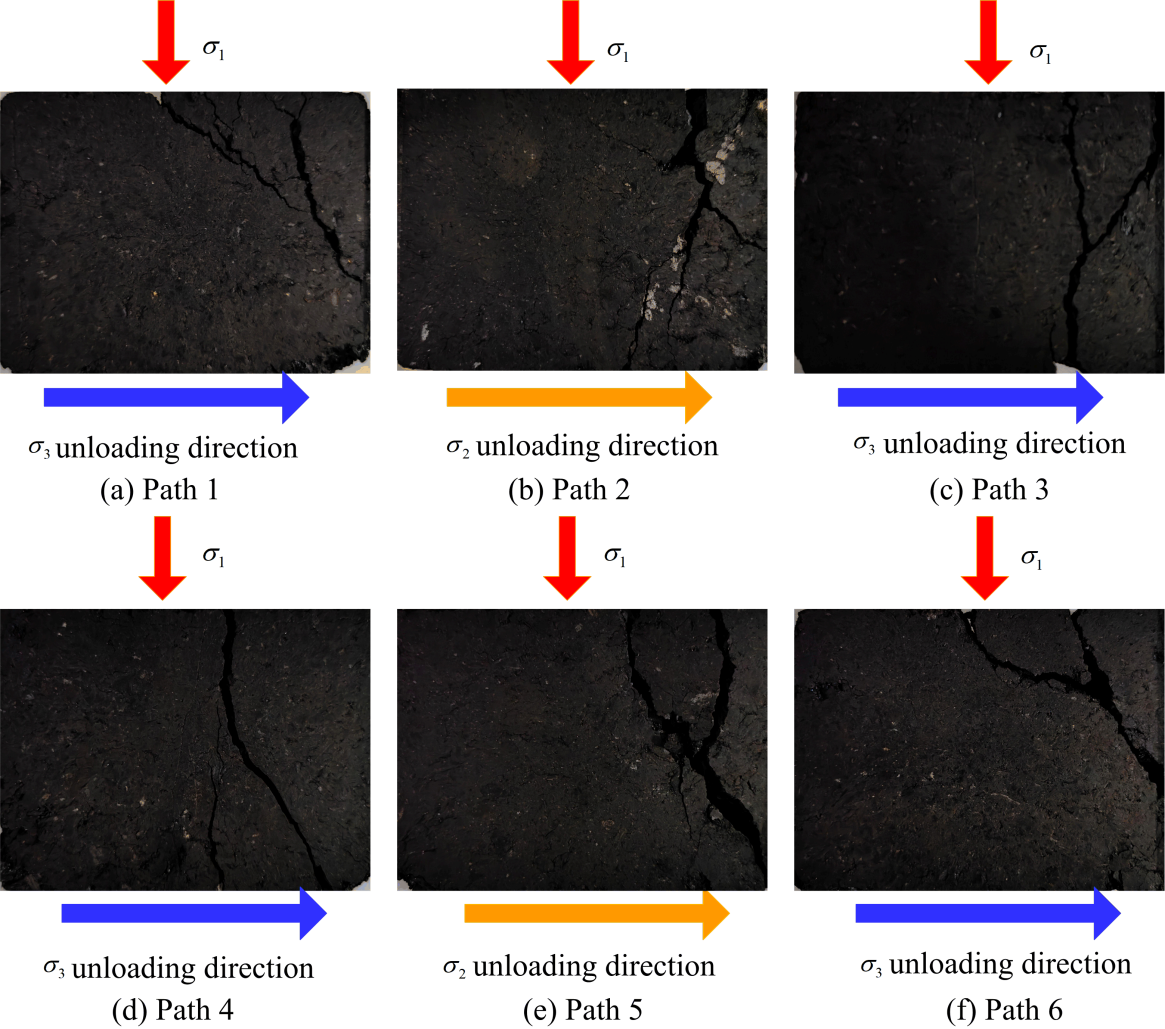
Fracture characteristics of coal under different stress paths. (a) Path 1. (b) Path 2. (c) Path 3. (d) Path 4. (e) Path 5. (f) Path 6.

The fracture characteristics of coal represent the ultimate manifestation of its failure process and serve as a crucial basis for elucidating its fracturing mechanisms. Fig 14 summarizes the fracture conditions of coal under six stress paths at the same stress level. As shown in the figure, coal undergoes axial compressive deformation and lateral expansion in the unloading direction under all six stress paths. As the loading and unloading test progresses, macroscopic cracks form and the coal fails in the unloading direction. For paths 1, 3, 4, and 6, coal fractures mainly occur in the $\sigma_{1}$ - $\sigma_{3}$ plane, with cracks generally forming a “Y” shape, indicating a compression-shear failure mode. Under paths 2 and 5, coal fractures mainly occur in the $\sigma_{1}$ - $\sigma_{2}$ plane, with significant lateral expansion and higher fracture degree, where the main crack also forms a “Y” shape accompanied by numerous microcracks, indicating a compression-shear and tension composite failure mode.

### 3.4 Acoustic emission activity of soft coal under complex stress paths

**Fig 15:**
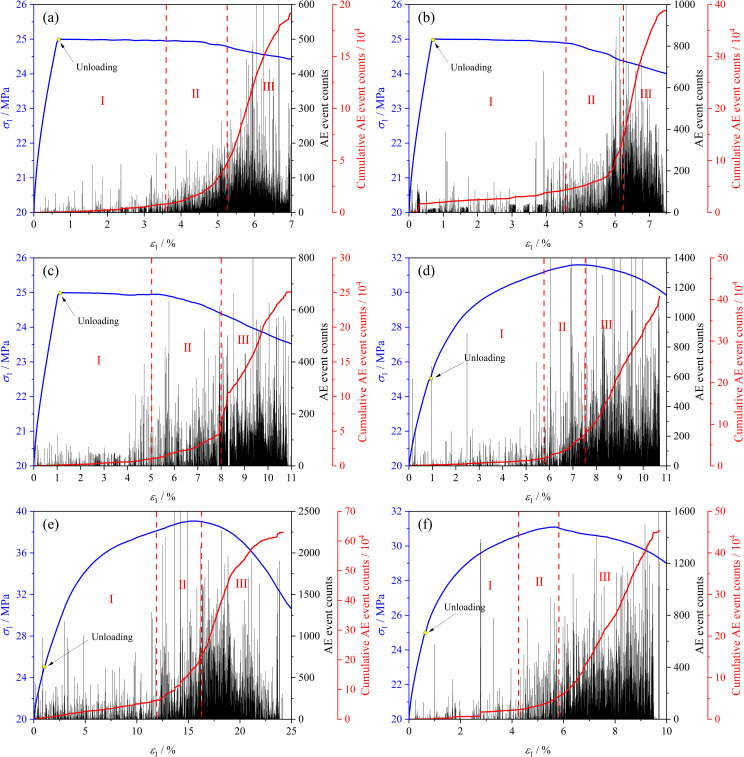
Evolution of acoustic emission of soft coal under different stress paths. (a) Path 1. (b) Path 2. (c) Path 3. (d) Path 4. (e) Path 5. (f) Path 6.

Monitoring the failure process of coal under complex stress paths using an acoustic emission system provides critical insights into the evolution of internal fractures and serves as a valuable basis for uncovering the underlying mechanisms of coal fracturing. Fig 15 shows the acoustic emission activity of coal during the loading and unloading process under different stress paths. It can be observed that the acoustic emission characteristics of soft coal are generally consistent across the six stress paths. From the unloading point, the acoustic emission process can be divided into three stages: stable period I, active period II, and explosive period III. In the early unloading stages of paths 1 to 3, the coal retains some load-bearing capacity with relatively few acoustic emission counts and a slow increase in cumulative acoustic emission counts. As the lateral stress decreases, the coal’s expansion rate increases, leading to gradual failure and instability. Acoustic emission counts become active, and the slope of the cumulative acoustic emission counts curve increases. As the lateral stress further decreases, the coal fully fails and loses load-bearing capacity, resulting in an explosive increase in cumulative acoustic emission counts. The early acoustic emission characteristics of paths 4 to 6 during loading and unloading are similar to paths 1 to 3. As loading and unloading progress, the slope of the coal’s stress-strain curve decreases, and acoustic emission counts become active. When the coal’s axial stress reaches its peak, acoustic emission counts surge until the coal fully fails and becomes unstable. The cumulative acoustic emission count curves under different stress paths show that the cumulative acoustic emission counts from highest to lowest are: Path 5>  Path 6>  Path 4>  Path 2>  Path 3>  Path 1. The cumulative acoustic emission counts of loading axial pressure and unloading confining pressure tests are overall higher than those of constant axial pressure and unloading confining pressure tests, indicating more severe internal coal fracturing during loading axial pressure and unloading. Under Path 5, the cumulative acoustic emission counts are significantly higher than other paths, primarily because Path 5 involves a conversion between $\sigma_{2}$ and $\sigma_{3}$, which induces a more complex crack evolution process within the coal. Specifically, in the early stages of unloading, the coal undergoes expansion deformation along the $\sigma_{3}$ direction. As the conversion between $\sigma_{2}$ and $\sigma_{3}$ occurs, the expansion direction gradually shifts toward $\sigma_{2}$, generating additional crack propagation during this process and resulting in increased acoustic emission activity.

## 4. Damage constitutive model of soft coal under complex stress paths

The damage constitutive model of soft coal describes the deformation and damage behavior of coal under external loads. Due to the complex porosity, fissures, and nonlinear mechanical properties of soft coal, establishing a reasonable damage constitutive relationship can accurately reflect the mechanical behavior of coal under true triaxial complex stress paths. This provides theoretical support for analyzing the stability of surrounding rock and designing support during soft coal roadway excavation.

Lemaitre’s [36] strain equivalence hypothesis suggests that the deformation caused by nominal stress *σ* acting on the damaged part of the coal is equal to the deformation caused by effective stress *σ*^′^ acting on the undamaged part. Combining this with the generalized Hooke’s law, we get:


$$\varepsilon_{i}=\frac{1}{E^{\prime }}[\sigma_{i}-\mu (\sigma_{j}+\sigma_{k})]=\frac{1}{E}[\sigma_{i}^{\prime }-\mu (\sigma_{j}^{\prime }+\sigma_{k}^{\prime })]$$ (11)

where $\varepsilon_{i}$ are the strains in the three principal stress directions (*i* = 1, 2, 3); $\sigma_{i}$, $\sigma_{j}$ and $\sigma_{k}$ are the principal stresses, and *i*, *j*, *k* can be 1, 2, 3. *E’* and *E* are the elastic moduli of the damaged and undamaged coal, respectively.

From Lemaitre’s strain hypothesis, we get:


$$\left[\sigma^{\prime }]=\frac{1}{1-D}[\sigma \right]$$ (12)

where $\left[\sigma^{\prime }\right]$ is the effective stress matrix and [σ] is the nominal stress matrix.

Combining Eqs (11) and (12), we get:


$$D=1-\frac{\sigma_{i}-\mu (\sigma_{j}+\sigma_{k})}{E\varepsilon_{i}}$$ (13)

Assuming the probability of micro-element damage in soft coal follows statistical laws and conforms to a two-parameter Weibull distribution, and assuming the micro-element strength of the coal is $F^{\prime }$, based on previous analysis, the Mogi-Coulomb criterion can describe the strength characteristics of soft coal under true triaxial complex stress paths. The micro-element strength $F^{\prime }$ is related to the octahedral shear stress $\tau_{\text{oct}}^{\prime }$, and can be expressed as:


$$F^{\prime }=\tau_{oct}^{\prime }+b\sigma_{m,2}^{\prime }$$ (14)

where $\tau_{\text{oct}}^{\prime }=\frac{\tau_{\text{oct}}}{1-D}$; $\sigma_{m,2}^{\prime }=\frac{\sigma_{m,2}}{1-D}$.

Substituting Eq (13) into Eq (14), we get:


$$F^{\prime }=\frac{(\tau_{oct}+b\sigma_{m,2})E\varepsilon_{i}}{\sigma_{i}-\mu \left(\sigma_{j}+\sigma_{k}\right)}$$ (15)

The damage probability density function can be expressed as:


$$P\left[F^{\prime }\right]=\frac{m}{F_{0}}{\left(\frac{F^{\prime }}{F_{0}}\right)}^{m-1}\exp\left[-{\left(\frac{F^{\prime }}{F_{0}}\right)}^{m}\right]$$ (16)

where *m* and $F_{0}$ are Weibull distribution parameters.

The damage probability density is a measure of the micro-element damage rate in coal. The gradual accumulation of micro-element damage leads to an increase in coal damage. The damage variable can be expressed as:


$$D=\int_{0}^{F^{\prime }}P(F^{\prime })=1-\exp\left[-{\left(\frac{F^{\prime }}{F_{o}}\right)}^{\text{m}}\right]$$ (17)

$$\sigma_{i}=E\varepsilon_{i}\exp\left[-{\left(\frac{F^{\prime }}{F_{0}}\right)}^{m}\right]+\mu \left(\sigma_{j}+\sigma_{k}\right)=E\varepsilon_{i}\exp\left[-{\left(\frac{(\tau_{oct}+b\sigma_{m,2})E\varepsilon_{i}}{F_{0}[\sigma_{i}-\mu (\sigma_{j}+\sigma_{k})]}\right)}^{m}\right]+\mu \left(\sigma_{j}+\sigma_{k}\right)$$ (18)

Transforming Eq (18) and taking the logarithm twice, we get:


$$\ln\left[-\ln\frac{\sigma_{i}-\mu \left(\sigma_{j}+\sigma_{k}\right)}{E\varepsilon_{i}}\right]=\ln F^{\prime }-m\ln F_{0}$$ (19)

Converting Eq (19) to a linear function, we get:


Y=mX+A (20)

Where,


$$\{\begin{array}{l} Y=\ln\left[-\ln\frac{\sigma_{i}-\mu \left(\sigma_{j}+\sigma_{k}\right)}{E\varepsilon_{i}}\right]\\ X=\ln F^{\prime }\\ A=-m\ln F_{0} \end{array}.$$ (21)

Substituting the test results under true triaxial loading and unloading paths into Eq (20), linear regression fitting was used, and in combination with Eq (21), the fitting parameters *m* and $F_{0}$ for the constitutive model under different stress paths were obtained, as shown in Table 3.

**Table 3:** Fitting parameters for different stress path tests.

Stress Path	Test Number	$\sigma_{1}$		$\sigma_{2}$		$\sigma_{3}$	
		*m*	*F*_0_/MPa	*m*	*F*_0_/MPa	*m*	*F*_0_/MPa
Path 1	C1-U3-2	\	\	\	\	0.4075	19.56
Path 2	C1-U2-2	\	\	0.7170	42.98	\	\
Path 3	C1-U2U3-2	\	\	0.3759	39.12	0.3961	18.62
Path 4	L1-U3-2	0.3273	17.07	\	\	0.3565	13.53
Path 5	L1-U2-2	0.3690	22.10	0.299	45.62	\	\
Path 6	L1-U2U3-2	0.3453	20.17	0.2689	47.35	0.3672	16.86

**Fig 16:**
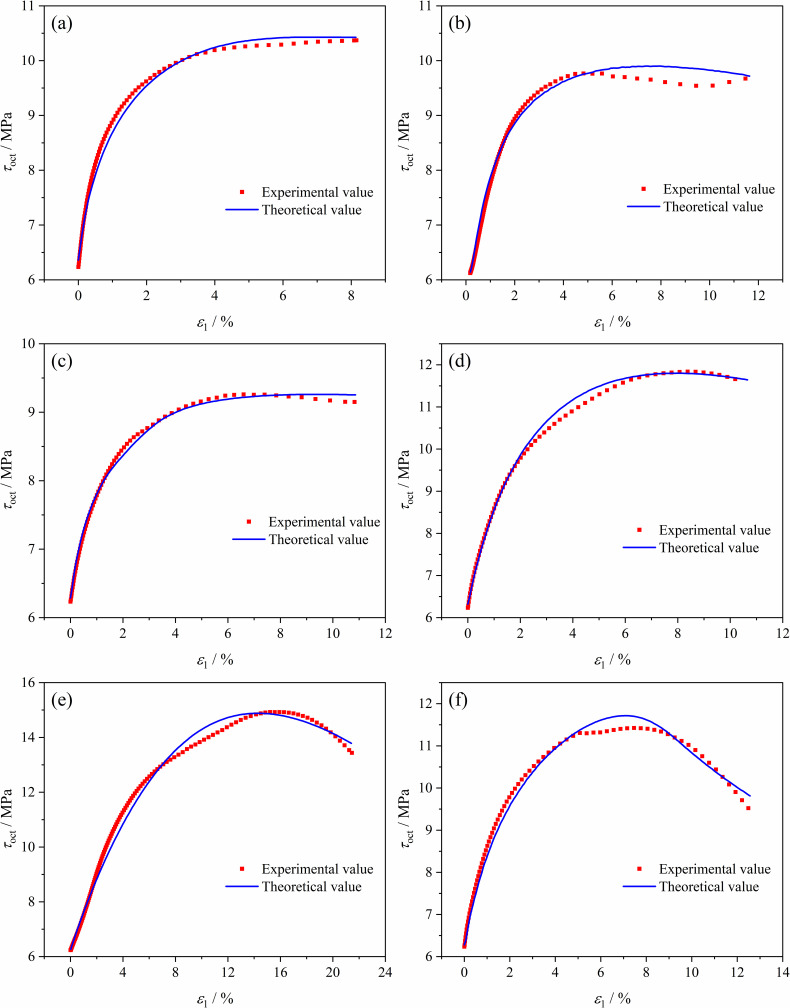
Theoretical and experimental $\tau_{\text{oct}}$ - $\varepsilon_{1}$ curves of coal under different stress path conditions. (a) Path 1. (b) Path 2. (c) Path 3. (d) Path 4. (e) Path 5. (f) Path 6.

Since the three principal stresses of soft coal vary under different stress paths, to uniformly verify the damage constitutive model of coal under different stress paths, the principal stress values were first calculated using the constitutive model of the coal, and the obtained principal stresses were then converted into the form of octahedral shear stress $\tau_{\text{oct}}$. The theoretical $\tau_{\text{oct}}$ - $\varepsilon_{1}$ curves were derived and compared with the experimental curves, as shown in Fig 16. The trend of the $\tau_{\text{oct}}$ - $\varepsilon_{1}$ experimental curves under different stress paths matches well with the theoretical curves, demonstrating the theoretical curves can accurately reflect the deformation strength characteristics of soft coal under true triaxial complex stress paths, proving the rationality of the damage constitutive model of soft coal.

Accurately predicting the deformation and failure behaviors of coal and rock under complex conditions is essential for ensuring the safety and stability of coal mining operations. This study presents some advancements in the damage constitutive modeling of soft coal when compared to previous models. Previous research on constitutive models has primarily focused on hard rock or coal subjected to single true triaxial loading paths. Although these studies offer valuable theoretical insights, their applicability to soft coal is limited. During roadway excavation, coal situated ahead of the tunnel face experiences complex stress conditions, including loading, unloading, and combined loading-unloading processes, resulting in a variety of stress paths. A single true triaxial loading path fails to adequately represent the mechanical behavior of coal under such conditions. Therefore, building on experimental results for soft coal under complex stress paths, this study employs the Mogi-Coulomb criterion to assess the strength of microelements. The developed damage constitutive model for soft coal provides a more precise depiction of damage evolution processes under complex stress paths. In practical engineering applications, the developed strength criterion and damage constitutive model for soft coal can be integrated with site-specific geological conditions to construct a mechanical model of soft coal roadways. This enables the derivation of analytical solutions for roof deformation and plastic zones under excavation-induced stress paths. The results can provide a more comprehensive and reliable theoretical basis for evaluating the stability of soft coal roadway roofs and optimizing support designs, offering significant value in improving excavation efficiency and preventing major accidents.

## 5. Conclusions

In this study, true triaxial loading and unloading tests were conducted under six different stress paths to investigate the deformation strength evolution, fracture characteristics, and acoustic emission activity of soft coal under complex true triaxial stress paths. Based on the test results, a damage constitutive model for soft coal under complex true triaxial stress paths was established. The main conclusions are as follows:

(1) The stress-strain curves of soft coal under the six stress paths can be divided into a stable stage (pre-peak stage) and a failure instability stage (post-peak failure instability stage). In the failure instability stage, the coal body undergoes rapid expansion deformation along the unloading direction and ultimately fails. The direction of expansion deformation varies under different paths: Paths 1, 3, 4, and 6 exhibit expansion and failure along the $\sigma_{3}$ direction, while Paths 2 and 5 exhibit expansion and failure along the $\sigma_{2}$ direction. In the constant axial pressure and unloading confining pressure tests, the coal is most prone to failure instability under Path 1 conditions. In the increasing axial pressure and unloading confining pressure tests, the coal is most prone to failure instability under Path 6 conditions.
(2) The strength characteristics of soft coal under complex true triaxial stress paths conform to the Mogi-Coulomb criterion, with a fitting degree *R*^2^ of 0.92, and the test data are mostly located on the theoretical yield surface. The evolution of the octahedral shear stress $\tau_{\text{oct}}$ under different stress paths shows that the octahedral shear stress $\tau_{\text{oct}}$ reaches its maximum value when the coal fails. The impact of stress paths on coal strength is significant, with the relationship being: Path 5>  Path 4>  Path 6>  Path 1>  Path 2>  Path 3.
(3) Regarding the fracture characteristics of the coal, under Path 1, Path 3, Path 4, and Path 6 conditions, the fractures mainly occur on the $\sigma_{1}$ - $\sigma_{3}$ plane, with the fracture mode being primarily compression-shear failure. Under Path 2 and Path 5 conditions, the fractures mainly occur on the $\sigma_{1}$ - $\sigma_{2}$ plane. Due to the conversion between $\sigma_{2}$ and $\sigma_{3}$ during the loading and unloading process, the direction of coal’s expansion deformation shifts, leading to a relatively higher fracture degree, with the fracture mode showing a combination of tension and compression-shear failure. In terms of acoustic emission activity, the soft coal’s acoustic emission process under the six stress paths can be divided into three stages: stable stage I, active stage II, and explosive stage III. The cumulative acoustic emission counts from highest to lowest are: Path 5>  Path 6>  Path 4>  Path 2>  Path 3>  Path 1.
(4) Based on the results of true triaxial complex stress path loading and unloading tests, a damage constitutive model for soft coal under complex true triaxial stress paths was established using damage mechanics and Weibull statistical distribution theory, incorporating the Mogi-Coulomb criterion to measure micro-element strength. Comparing the theoretical model curves with the test curves revealed that the theoretical curves can accurately reflect the deformation strength characteristics of soft coal under complex true triaxial stress paths, validating the model’s rationality. The model provides a crucial theoretical foundation for enhancing predictions and evaluations of roadway roof stability in soft coal seams.
